# Visual Hallucinations in the Psychosis Spectrum and Comparative Information From Neurodegenerative Disorders and Eye Disease

**DOI:** 10.1093/schbul/sbu036

**Published:** 2014-06-13

**Authors:** Flavie Waters, Daniel Collerton, Dominic H. ffytche, Renaud Jardri, Delphine Pins, Robert Dudley, Jan Dirk Blom, Urs Peter Mosimann, Frank Eperjesi, Stephen Ford, Frank Larøi

**Affiliations:** ^1^Clinical Research Centre, Graylands Hospital, North Metropolitan Health Service Mental Health, Perth, Western Australia, Australia;; ^2^Centre for Clinical Research in Neuropsychiatry, School of Psychiatry and Clinical Neurosciences, the University of Western Australia, Perth, Western Australia, Australia;; ^3^Northumberland, Tyne and Wear NHS Foundation Trust, Bensham Hospital, Gateshead and Newcastle University, Newcastle Upon Tyne, UK;; ^4^Institute of Psychiatry, King’s College London, London, UK;; ^5^Laboratoire de Neurosciences Fonctionnelles & Pathologies, Université Droit & Santé (UDSL), Univ Lille Nord de France and Centre Hospitalier Universitaire (CHU Lille), Hôpital Fontan, Lille, France;; ^6^School of Psychology, Newcastle University, Newcastle Upon Tyne, UK;; ^7^South of Tyne Early Intervention in Psychosis Service, Northumberland, Tyne and Wear NHS Foundation Trust, Newcastle Upon Tyne, UK;; ^8^Parnassia Psychiatric Institute, The Hague, The Netherlands;; ^9^Department of Psychiatry, University of Groningen, Groningen, The Netherlands;; ^10^University Hospital of Old Age Psychiatry, University of Bern, Bern, Switzerland;; ^11^Ophthalmic Research Group, School of Life and Health Sciences, Aston University, Birmingham, UK;; ^12^Department of Psychiatry, Sir Charles Gairdner Hospital, North Metropolitan Health Service Mental Health – Older Adult Program, Perth, Western Australia, Australia;; ^13^Department of Psychology: Cognition and Behaviour, University of Liège, Liège, Belgium

**Keywords:** visual hallucinations, schizophrenia, psychosis, cognition, imaging

## Abstract

Much of the research on visual hallucinations (VHs) has been conducted in the context of eye disease and neurodegenerative conditions, but little is known about these phenomena in psychiatric and nonclinical populations. The purpose of this article is to bring together current knowledge regarding VHs in the psychosis phenotype and contrast this data with the literature drawn from neurodegenerative disorders and eye disease. The evidence challenges the traditional views that VHs are atypical or uncommon in psychosis. The weighted mean for VHs is 27% in schizophrenia, 15% in affective psychosis, and 7.3% in the general community. VHs are linked to a more severe psychopathological profile and less favorable outcome in psychosis and neurodegenerative conditions. VHs typically co-occur with auditory hallucinations, suggesting a common etiological cause. VHs in psychosis are also remarkably complex, negative in content, and are interpreted to have personal relevance. The cognitive mechanisms of VHs in psychosis have rarely been investigated, but existing studies point to source-monitoring deficits and distortions in top-down mechanisms, although evidence for visual processing deficits, which feature strongly in the organic literature, is lacking. Brain imaging studies point to the activation of visual cortex during hallucinations on a background of structural and connectivity changes within wider brain networks. The relationship between VHs in psychosis, eye disease, and neurodegeneration remains unclear, although the pattern of similarities and differences described in this review suggests that comparative studies may have potentially important clinical and theoretical implications.

## Introduction

Hallucinations are defined in different ways by different philosophical traditions.^1^ In the clinical domain, a visual hallucination (VH) is a visual percept, experienced when awake, which is not elicited by an external stimulus. It contrasts with a visual illusion which is elicited by an external stimulus but differs from the percept normally associated with the stimulus. VHs occur in a wide-range of organic and psychiatric conditions, as well as in the absence of any demonstrable pathology. These experiences have been well described and researched in the context of organic disorders, particularly eye disease and neurodegenerative conditions such as dementia with Lewy bodies (DLB) and Parkinson’s disease (PD), but VHs have been largely neglected in psychiatric disorders and delirious states and in nonclinical populations.

Although the diagnostic manuals for mental disorders list hallucinations as a primary characteristic symptom in psychotic disorders, *auditory* hallucinations are the symptoms that clinicians commonly ask about. One explanation lies in the traditional beliefs that VHs are more common in organic states than in psychosis.^2^ It is also often difficult to decide whether the full criteria for the presence of VHs have been fulfilled when a range of other perceptual abnormalities are reported.

In this article, we review the available evidence with regards to the prevalence, phenomenology, clinical characteristics, and assessment methods for VHs in the psychosis spectrum alongside studies of cognition, brain imaging, electrophysiology, and treatment (cognitive behavioral and pharmacological). Given the lack of available literature on other psychiatric disorders, our focus is on schizophrenia, affective psychosis (ie, bipolar and depressive disorders), and nonclinical population groups (who experience VHs outside of the context of any psychiatric or somatic disease). The psychosis evidence is compared with evidence related to eye and neurodegenerative disease in the hope that similarities and differences between different clinical contexts will help our understanding of the underlying mechanisms of VH and its treatment.

To our knowledge, this is the first review of VH in psychosis. For the first time, a cross-disciplinary and cross-diagnostic examination of VH is also presented. Our broad aim is to provide a useful base for future studies on the topic and specifically to achieve a clearer understanding of VH in psychiatric illness and greater clarity regarding the assessment of VH for use in clinical practice and research.

Parts of this article were presented in Durham at the 2nd International Consortium on Hallucination Research (ICHR) 2013 conference.^3^

## Epidemiology

### Psychosis

The centrality of VH in psychosis has been put into question by studies which show that auditory, and not visual, hallucinations are among the cardinal symptoms of schizophrenia which are common in all cultures.^4^ VHs, however, appear to be more frequent in schizophrenia than commonly thought.

Table 1 describes studies that have provided the point prevalence of VH in schizophrenia. Estimates vary widely from 4% to 65%, reflecting variations in population and ascertainment methods. Out of 29 studies that have addressed this issue (5873 participants), the weighted mean prevalence of VH in schizophrenia is 27% (SD = 9). For comparison, the weighted mean of auditory hallucinations as provided by the same studies is 59% (range: 25%–86%; SD *=* 15), ie, twice as frequent as VH, although it is possible that clinician biases toward auditory hallucinations have skewed the results.

**Table 1.** The Comparative Point Prevalence of Visual and Auditory Hallucinations in Schizophrenia

Authors	*n*	Modality of Hallucinations	
		Visual (%)	Auditory (%)
Bowman and Raymond (1931)^ 5 ^	1408	22	53
Arnold (1949)^ 6 ^	^a^	14	78
Feinberg (1962)^ 7 ^	19	4	84
Malitz et al (1962)^ 8 ^	100	9	50
Vitols et al (1963)^ 9 ^	110	13	35
Goldberg et al (1965)^ 10 ^	270	18	45
Mott et al (1965)^ 11 ^	50	24	66
Small et al (1966)^ 12 ^	50	30	66
Chapman (1966)^ 13 ^	^a^	40	^a^
Holmboe and Astrup (1967)^ 14 ^	255	25	79
Jansson (1968)^ 15 ^	293	13	25
Goodwin et al (1971)^ 16 ^	45	59	82
Eggers (1973)^ 17 ^	^a^	44	71
McCabe (1976)^ 18 ^	40	20	36
Young (1974)^ 19 ^	20	45	^a^
Zarroug (1975)^ 20 ^	69	47	62
Ciompi and Müller (1976)^ 21 ^	^a^	18	58
McCabe (1976)^ 18 ^	25	20	52
Deiker and Chambers (1978)^ 22 ^	28	64	86
Huber (1979)^ 23 ^	^a^	33	75
Ndetei and Singh (1983)^ 24 ^	51	43	43
Ndetei (1984)^ 25 ^	141	15	41
Winokur et al (1985)^ 26 ^	140	32	78
Phillipson and Harris (1985)^ 27 ^	73	62	44
Bracha et al (1989)^ 28 ^	43	56	42
Owens and Slade (1989)^ 29 ^	^a^	29	63
Mueser et al (1990)^ 30 ^	117	14	71
Jablensky et al (1992)^ 4 ^	1288	30	55
Bauer et al (2011)^ 31 ^	1238	34	79
Total: 29 studies, *n* = 5873 participants		Weighted mean = 27%	Weighted mean = 59%
		SD = 9.73	SD = 15.30

*Note*: ^a^Not assessed or missing data.

Psychotic symptoms such as hallucinations and delusions are also a common feature of affective disorders including bipolar disorder.^6^ Table 2 shows that out of 12 studies that differentiated between different modalities of hallucinations, the weighted mean frequency of VH is approximately 15% (range: 6%–27%, SD *=* 9). Similarly to schizophrenia, the rates of VH in bipolar disorder are approximately half that of auditory hallucinations (28%).

**Table 2.** The Comparative Prevalence of Visual Hallucinations and Auditory Hallucinations in Bipolar and Affective Disorder

Authors	*n*	Diagnosis	Modality of Hallucinations	
			Visual (%)	Auditory (%)
Bowman and Raymond (1931)^ 5 ^	1009	Mania	9	17
Rosenthal et al (1966)^ 32 ^	79	Mania	21	30
Winokur (1969)^ 26 ^	100	Mania	9	21
Goodwin et al (1971)^ 16 ^	28	Primary affective disorder	72	82
Taylor and Abrams (1975)^ 33 ^	52	Mania	23	47
Rosenthal et al (1980)^ 34 ^	32	Mania	25	30
Winokur (1984)^ 26 ^	122	Bipolar disorder	9	14
Black and Nasrallah (1989)^ 35 ^	467	Acute bipolar disorder	6	13
Mueser et al (1990)^ 30 ^		bipolar disorder	25	75
	37	Severe affective disorder	10	17
Keck et al (2003)^ 36 ^	352	Mania	22	25
Baethge et al (2005)^ 37 ^	33	Manic/mixed type with hallucinations	27	54
	32	Depressed type with hallucinations	25	59
Tillman et al (2008)^ 38 ^	549	BPD	26	57
Total: 12 studies, *n* = 2892			Weighted mean = 15%	Weighted mean = 28%
			SD = 9.75	SD = 18.11

*Note*: BPD = bipolar disorder.

Overall, these data challenge the assumption that VHs are atypical or uncommon in psychosis.

### Subclinical Symptoms in the General Community

Occasional hallucinatory experiences are fairly common in community-living individuals. However, community and epidemiological studies rarely report the prevalence rates for VH specifically and independently from other hallucinatory experiences, with few exceptions. Table 3 (A) shows that the weighted mean for VH in the general community is 7.3% (SD = 5, based on 6 studies).

**Table 3.** The Comparative Prevalence of Visual Hallucinations and Auditory Hallucinations in the General Community (A) and After Excluding Hallucinations Arising From Drug-Taking or Physical Illness (B)

	*n*	Visual Hallucinations (%)
(A) General population		
Eaton et al (1991)^ 39 ^	810	8
Tien (1991)^ 40 ^	18572	14
Ohayon (2000)^ 41 ^	13057	3.2
Waters et al (2003)^ 42 ^	562 (university students)	10
Larøi and Van der Linden (2005)^ 43 ^	236 (university students)	32
Kessler et al (2005)^ 44 ^	9282	6.3
	Total: 6 studies, *n* = 42519	Weighted mean = 7.35%
		SD = 5.08
(B) Excl drug-taking and physical illness		
Eaton et al (1991)^ 39 ^	810	4
Tien (1991)^ 40 ^	18572	7
Van Os et al (2000)^ 45 ^	7076	2–6
	Total: 3 studies, *n* = 26 458 participants	Weighted mean = 6%
		SD: 1.73

Little is known about what distinguishes visual hallucinators from non-hallucinators in the general population, but evidence suggests that factors such as intoxication and withdrawal from substances such as alcohol, cannabis and cocaine, and other physical states such as physical illness and stress are linked to VH.^46,47^ Importantly, when research criteria exclude hallucinations arising from drug-taking or physical illness, the weighted percentages of hallucinations in the community reduce to 6% (see table 3, B).

### Eye Disease and Neurodegenerative Conditions

For comparison, it is helpful to understand the epidemiology of VH in other disorders in which VHs are common. The frequency estimates for VH in PD range from 15% to 40% (comparable to those in psychosis), and the frequency roughly doubles for PD dementia (PDD) (30%–90%) and DLB (60%–90%).^48,49^ In age-related eye disease, between 10% and 60% of patients experience complex VH, depending on the severity of visual loss. The most common eye disease associated with VH is age-related macular degeneration.^50,51^ VHs are the diagnostic criterion for Charles Bonnet syndrome (CBS). Such observations have prompted many authors to suggest that VH arise because of dysfunctions involving visual processing.

## Demographic and Clinical Characteristics

### Gender

Contradictory data have emerged regarding a VH gender pattern in psychosis or organic illness.^5,37,52,53^ By contrast, studies in PDD and DLB do not report different frequencies of VH by gender, although it is important to consider the gender ratio in these disorders which are more common in men than women.

### Age Risk Factor and Longitudinal Course

With regards to age as a risk factor for VH in psychosis, some studies have demonstrated evidence that VH are more common in younger, compared with older, individuals with schizophrenia,^31,53^ although negative findings exist.^16^ A similar inconsistency is found in the eye disease literature with only 3 of 9 studies investigating age and VHs reporting a weak association.^51^

By contrast, in nonclinical populations, the prevalence of VH is maximal in adolescence, and late adulthood, between which times the frequencies decrease.^40,54^ One explanation for increased frequency of VH with age is that the prevalence of most chronic disorders increases with age, as does the prevalence of neurodegenerative disorders and age-related eye disease. In further support, the prevalence of death-bed visions among the dying may be as high as 50%.^55^

Thus, there appears to be a bimodal distribution of VH in the population with one peak in late adolescence and early adulthood, which is associated with psychosis, and a second increase in late life associated with eye and brain disease.

With regards to the longitudinal course, it is believed that hallucinations in schizophrenia tend to decrease as individuals age,^56^ although it is important to note that the risk of schizophrenia also declines with age.

### Clinical Characteristics of VH

The presence of VH in psychosis has often been linked to a more severe psychopathological profile^30,57^ and to a less favorable prognosis.^18^ In patients with bipolar disorder, Baethge et al^37^ found that hospitalization for individuals with hallucinations (all modalities) averaged 17% longer than those who did not hallucinate.

In the general community, a link between VH and anxiety (OR = 5.0)^41,58^ and psychotic pathology (OR = 6.6)^41^ has been reported. The important role of negative emotions in VH is illustrated in studies showing that stress and bereavement are linked to VH. For example, hallucinations of a dead spouse are common grief reactions in older adults.^59^

The literature in neurodegenerative disease is consistent with this view of greater psychopathology in VH, with studies showing poorer response to treatment, greater mortality and morbidity, major depressive syndrome, and a move to institutional care.^60,61^

Thus, in both younger patients with psychosis and older patients with neurodegenerative disease, VHs are associated with poorer functioning and outcome. The same is not true of eye disease (eg, CBS) where the symptoms improve over time without progressive loss of cognitive function.

### Association With Other Symptoms

In schizophrenia, VHs typically co-occur in association with other hallucinations and other sensory modalities.^16,28,30,62^ For example, it has been reported that co-occurring visual and auditory hallucinations occur in up to 84% of individuals with schizophrenia.^30^ Furthermore, early evidence suggested VH never occurred in psychosis without the presence of auditory hallucinations (either at the same time—multimodality hallucinations—or on different occasions).^62^ In Oorschot et al’s^57^ sample of individuals with mixed psychosis, VHs occurring alone were rarer than auditory hallucinations occurring alone. As shown in table 1, auditory hallucinations predominate over VH in terms of their relative prevalence.

Hallucinations in multiple modalities have also been noted in individuals with severe depression^63^ and with mixed psychiatric diagnoses,^64^ as well as in nonclinical adults and adolescents (*n* = 355, 11–13 years).^65^ Such co-occurrences of auditory/VHs suggest a common hallucinatory mechanism which, in combination with specific sensory dysfunctions, determines the modality of hallucinations.

It is important to note, however, that “simultaneous” (or “fused”) auditory and VHs are not a frequent occurrence.^16^ In most cases, they are experienced at different times (eg, an auditory hallucination one day and a VH the next). Furthermore, when simultaneous auditory/VHs do occur, they are typically unrelated^66^ (eg, seeing the devil while hearing the voice of a relative inside one’s head), suggesting that the mechanisms for auditory and VHs in these disorders must be partly independent, though with some overlap.

By contrast, a different pattern can be observed in organic disease. In PD, VHs predominate over auditory hallucinations,^67^ and in CBS, the absence of hallucinations in other modalities is a diagnostic criterion. Where eye disease co-occurs with dementia, auditory hallucinations and delusions may appear as the dementia progresses, as is the case in PD^68^ or dementia alone.^69^ In these disorders, auditory and VHs very rarely occur at the same time.

Overall, the relative proportion of auditory to visual modality hallucinations seems to differentiate the psychosis spectrum from organic conditions.

## Phenomenological Features of VH

### Psychosis

There are few systematic or comprehensive studies of VH as dimensional experiences in psychosis. Exceptions include Goodwin et al,^16^ Gauntlett and Kuipers,^64^ and Dudley et al.^70^ For the following review, information was drawn from these and other studies.^12,16,28,53,62,71,72^

#### Perceptual Qualities.

VHs in psychosis are reported to have the physical properties of real perceptions. They are often life-sized, detailed, and solid. They are projected into the external world in most cases and are typically “anchored in external space,” either just beyond the reach of individuals or further away. VHs often have 3 dimensional shapes, with depth and shadows, and distinct edges. VH can be colorful or in black and white. Images are often described as dynamic with the contents changing in size, shape, and movement but may also be static.

#### Temporal Aspects.

The frequency of hallucinatory episodes varies from rare to frequent, and episodes can last several seconds to several minutes. VH may be experienced during both day and night.

#### Content.

Fully formed VHs are more common than unformed visual experiences and distortions. Complex VHs often comprise images of people (walking, family), faces, animals, objects, or events (eg, visions of fires) taking place in front of the individual. In contrast to hallucinations in organic conditions, a common theme is of visions with frightening content (bugs, dogs, snakes, distorted faces), and these are linked to distress. Unlike VH in PD, visions of dead people are rare, although visions of God, angels, the devil, saints, and fairies are common.

#### Reality.

VHs are perceived to be real and “definitely present” in a concrete sense. In further support for the subjective reality of the experience, a majority of individuals undertake some activity directly related to the vision—such as moving toward the vision, hitting at the vision, or moving away. In the case of distressing images, individuals may act to keep themselves safe.

#### Sense of Control.

A lack of control over the content and appearance is a prominent feature of VH in psychosis. Individuals are surprised when VHs occur and are generally helpless to change or stop them. In general, individuals believed that visions are experienced only by themselves.

#### Onset and Triggers.

The onset of VH has been linked to stress, tiredness, loneliness, and relationship problems in psychiatric individuals. Other psychological triggers include negative emotions, and they may co-vary with levels of anxiety.

#### Reactions.

A mixture of positive and negative images may be experienced. Reactions vary and can include pleasure, happiness and reassurance, or indifference. Images can also evoke fright, depression, sadness, or hopelessness. While the *content* of VH is often impersonal or unrelated to the person, images are often interpreted to have personal implications. Many individuals believe that the content of visions must be acted upon, for fear of a negative event occurring as a consequence. This differs from hallucinations in organic conditions, where VHs are not generally perceived to be real.

#### Beliefs and Appraisals.

VHs may be believed to arise from an external source, particularly of supernatural origin. Images of powerful religious figures are often perceived to be visions intended as a sign for the person to act and as a threat to their physical or psychological wellbeing. This can also impact on the individual’s belief system and can be used as evidence for their delusional beliefs.

Overall, such rich phenomenology of VH in psychosis points to true hallucinatory experiences and not only misperceptions. This close examination of VH characteristics also reveals remarkable similarities with auditory hallucinations with regards perceptual quality, contents, lack of control, beliefs, appraisals, and reactions.

With regards a comparison with organic disorders, broadly speaking, the form and character of VH in psychosis is similar to those seen in eye disease and neurodegeneration. Although there is a notable lack of studies making direct comparisons, there are several characteristics that distinguish VH in psychotic disorders from other disorders, namely frightening contents, emotional reactions, and appraisals of personal significance; a lack of illusions (common in neurodegenerative disease) and simple VHs (common in eye disease) also differ.

### Subclinical Symptoms in the General Community

Nonclinical individuals in the community report vivid VH, where objects are real and uncontrollable, but often of brief duration.^73^ The features of VH in nonclinical samples are broad-ranging and include unformed VH (such as dots, flashes of light and other unformed shapes) and formed VH (people and faces, but also aliens).^65^ In contrast to VH in psychosis, hallucinated images in nonclinical populations are less likely to involve complex scenes but more likely to show people.^52^

These VH clinical features are similar to that of auditory hallucinations in nonclinical populations, which also tend to be brief and mostly neutral or pleasant in content.^74^

### Eye Disease and Neurodegenerative Conditions

For comparison with the above, the most detailed accounts of visual content are found in the eye disease literature (CBS).^75^ The commonest contents are of simple hallucinations, followed by images of people or animals (50%–80%), patterns (55%), faces often described as grotesque and cartoon-like (40%), landscapes or inanimate objects (20%). They are usually colorful. Unlike in psychosis, VHs in CBS are not generally perceived to be real or to have personal meaning. Only in a third of the cases is the content negative. Once individuals understand they are a common feature of poor vision, anxiety about VH generally decreases. This differs from psychosis, where lack of insight may contribute to distress about these experiences. Other dimensional features related to clarity, frequency, temporal factors, and appearance are similar to those in psychosis.^76^ In PD, VHs mostly consist of people who may be familiar or not, and alive or deceased (73%), animals (33%), and objects (19%) which may appear briefly and move sideways (passage hallucination).^49^ The contents are fairly stereotyped and repetitive but may last for hours at a time. Experiences appear to be real and outside voluntary control but are not often perceived as frightening. In dementia, VHs tend to be of fairly mundane content, including unfamiliar or small figures, faces, animals, and objects/machines, and are rarely distressing.^69^

**Fig. 1.**
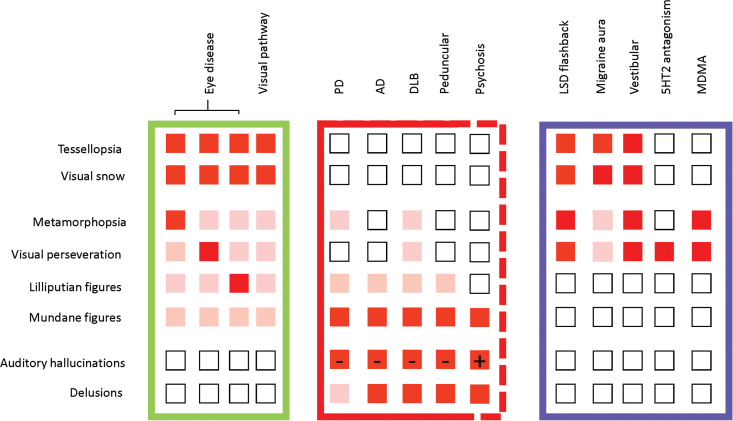
Visual perceptual symptoms and their clinical contexts.^77^ A range of clinical conditions (columns) are cross-tabulated with visual hallucination (VH) content and related phenomena (rows). For each condition, the percentage of individuals with VHs reporting a given content is coded red (>20%), pink (10%–20%), or white (not reported or < 10%). The prevalence of each symptom in psychosis is taken from.^64^ For auditory hallucinations, (+) indicates higher prevalence than VH and (−) indicates lower prevalence than VH (figure adapted from ffytche^77^). Visual experiences in schizophrenia best match the phenomena reported in the red box derived from PD, AD, DLB, and peduncular lesions—but not the green (eye and visual pathway pathology) and blue (serotonergic syndrome) boxes.

Figure 1 (adapted from ffytche^77^) provides an overview of VH in different clinical settings, where a range of clinical conditions (columns) are cross-tabulated with VH content and related phenomena (rows). Comparing the phenomenology of VH in psychosis with other conditions suggests that they are most similar to VH in cortical and subcortical disorders (red box) but differ in the relative predominance of auditory and VHs. Whether this is an important neurobiological distinction is unclear, and the boundary of the grouping has been left open as indicated by the dashed line.

## The Assessment of VHs

It is often difficult to distinguish VH from related visual phenomena such as illusions, distortions, and misperceptions that co-occur with VH in psychosis and other conditions. The relationship between what is seen and what is actually present in the environment is complex and poorly understood with internal models and top-down inference interacting with visual inputs. The match between what is perceived and what is present is closest for veridical perception (our day-to-day visual perceptual experience), tenuous for illusions, and absent for hallucinations; however, there are no sharp dividing lines between categories. Based on a working definition of hallucinations,^78^ the key features to elicit in an assessment of VH are (see Box 1):

1. They must be experienced in full consciousness (rules out sleep-related VH, fever, delirium, and hypnosis).
2. Not elicited by an external stimulus (excludes visual distortions and illusions).
3. The experience has a sense of reality to resemble a veridical perception (must have physical properties of real perceptions and be located in external space; note that this does not mean that VH must be perceived to be real or that insight must be lacking).
4. The subject does not feel s/he has direct and voluntary control (rules out visual imagery).

**Box 1. A Diagnostic Algorithm for Visual Hallucinations and Related Phenomena^79^**

**Question 1: Are the visual phenomena experienced (a) during sleep, (b) on the border of waking and sleeping, or (c) while awake?**

a) During sleep
  Rule out: dream
  Possibility 1: incubus phenomenon
  Possibility 2: visual sleep start
b) On the border of waking and sleeping
  Possibility 1: hypnagogic hallucination
  Possibility 2: hypnopompic hallucination
c) While awake
  Go to Question 2

**Question 2: Are the visual phenomena perceptual in nature? (ie, with physical properties comparable with sensory perceptions)**

No   Rule out: imagery
Yes   Continue with Question 3

**Question 3: Do they constitute (a) images without a representation in the outside world, (b) images with a representation in the outside world, or (c) distortions?**

a) Without representation in the outside world (visual hallucinations):
   Go to Question 4
b) With a representation in the outside world (visual illusions):
   Go to Question 5
Distortions (metamorphopsias):
   Go to Question 6

**Question 4: Are the hallucinations (a) simple in nature, (b) geometric, (c) complex, or (d) compound (ie, multimodal) in nature?**

a) Simple hallucinations
  Rule out: physiological phenomena such as blue-field entoptic phenomenon, macular star pattern, or mouches volantes (uncomplicated)
  Determine: type and context
b) Geometric hallucinations
  Determine: type and context
c) Complex hallucinations
  Determine: type and context
d) Compound hallucinations
  Determine: type (ie, sensory modalities) and context

**Question 5: Are the illusions attributable to (a) physical reality, (b) the perceptual system, or (c) mental associations?**

a) Physical illusions
  Determine: type and context (never pathological!)
b) Physiological illusions
  Determine: type and context
c) Cognitive illusions
  Determine: type and context

**Question 6: Do the distortions have (a) a peripheral or (b) a central origin?**

a) Peripheral metamorphopsias
  Determine: type and context
b) Central metamorphopsias
  Determine: type and context

## Cognition

Cognitive processes of VH in psychosis have rarely been investigated. In organic disorders, hallucinations are held to reflect disorders within distributed perceptual systems. The Perceptual and Attentional Deficit model advocates for a combination of impaired visual processing and attention in VH in neurodegenerative disorders and eye disease.^80^ Diederich et al’s^81^ contemporaneous PD-developed interactive model similarly proposes a combination of poor visual input and processing with defective central visual monitoring. More recently, Shine et al’s Attentional model^82^ proposes that hallucinations occur in PD when there is underactivity in dorsal attentional networks in the presence of ambiguous percepts. Each of these models, therefore, proposes that combined attentional and visual perceptual problems lead to VH.

In psychosis, however, perceptual processes are usually believed to be intact or even overactive. It has been suggested that VH in psychosis may be best understood in terms of the intrusion into awareness of subconscious images or highly vivid mental imagery, as modulated by personal, social, or psychological variables.^58^ In support, an increase in the salience of imagery has been found in schizophrenia individuals with VH.^45^ Other cognitive deficits found in psychotic VH include source-monitoring impairments, such as difficulties making internal/external and self/other discriminations^83^ and confusion regarding whether the source of material was real or imagined.^84^ Such source-monitoring deficits are also commonly found in auditory hallucinations^85^ and may lead to these events being believed as real.

Personal, social, and cultural contributors to VH have not yet been systematically investigated, although top-down mechanisms (which include expectations, personal meaning-making, and social context) may influence dynamic attentional processes by biasing the competition between potential perceptions toward objects and persons that have personal and social relevance. In support for this view, stress and bereavement are linked to the onset of VH. Further support comes from cognitive models of psychosis^78^ that propose that key influences of personal events, social/cultural context, and expectations have the ability to activate percepts in the absence of external stimulation.

Altogether, mechanisms of VH in psychosis are not well understood. Interesting similarities with the literature on auditory hallucination exist, including source-monitoring deficits and the strong influence of top-down mechanisms. By contrast, evidence for visual processing difficulties which feature strongly in models of VH in the organic literature^77^ is lacking.

## Brain Imaging and Electrophysiology

In the field of psychosis, a study conducted in individuals with first-episode psychosis (*n* = 15 with VH) showed per-hallucinatory activity within visual association cortex, while the vividness of VH was linked to activity within the primary visual cortex.^86^ Activity in the visual association cortex during VH has also been demonstrated in single case studies in schizophrenia.^87,88^ Concerning the organic literature, dysfunctions of higher visual processing areas during or between VH episodes have been found in Alzheimer’s disease,^89^ DLB,^90,91^ and CBS^92^ within the ventral and the dorsal visual pathways involved in object recognition and visuospatial memory^93,94^ as well as the frontal lobes.^95^ Furthermore, structural changes in these areas were independent of cognitive function and age.^96^ Overall, symptom-capture studies in these populations showed that the location of the per-VH activity correlated with the phenomenological content of the hallucinatory experiences (ie, color-specialized cortex if in color, face-specialized cortex if faces are experienced, etc).

Connectivity studies have much relevance for the study of VH, given that dysconnectivity is a prominent model for psychosis, which could also apply to specific symptoms such as hallucinations.^97^ The dysconnectivity hypothesis suggests that the existence of impaired connectivity between different brain regions is responsible for abnormal functional integration within neural networks. In support for dysconnectivity, using diffusion tensor imaging, Ashtari and collaborators showed that adolescents suffering from early-onset schizophrenia with a history of VH exhibited lower fractional anisotropy, potentially reflecting a loss in white matter integrity, in the left inferior longitudinal fasciculus which connects temporal and occipital cortices, when compared with individuals without VH.^98^

The hippocampal complex (HC) is also of considerable interest with regards to VH in both neurodegeneration and psychosis. The HC has been shown to be atrophied in individuals with PD and VH,^99^ and it appears to occupy a key place within the networks involved in VH in psychosis.^100^ For example, a recent multimodal connectivity study, combining functional connectivity, tract-based spatial statistics, and shape analysis of the HC, confirmed differential connectivity patterns of this structure in individuals with schizophrenia. Moreover, this abnormal connectivity depends on the sensory-modality of hallucinatory experiences involved and was particularly obvious with visual areas in patients with VH.^97^ In addition, a symptom-capture study of VH in schizophrenia^87^ reported cortical activity in the HC, although this was not reported in CBS.^92^

A particular subtype of functional connectivity studies focuses on spontaneous fluctuations at rest. It has already been used in the field of auditory hallucinations to test the hypothesis of disrupted intrinsic connectivity as a pathological mechanism.^101^ Jardri and colleagues’ study confirmed a dynamic interaction between association sensory cortices and the default-mode network (DMN) for patients with multisensory hallucinations, including VH.^86^ Sensory and DMN networks were found to be anticorrelated during the experience of hallucinations; furthermore, the DMN spatial and temporal instability persisted during non-hallucinatory periods.^86^ Impaired interactions with the DMN have also been suggested in the pathophysiology of VH in PD,^82^ but contrary to what has been proposed in first-episode psychosis in which the DMN seems primarily and intrinsically affected, these authors proposed an external DMN interference through aberrant interactions with ventral and dorsal attentional networks.

Fewer studies have investigated the electrophysiology of VH. Spencer and colleagues found reduced visual cortex activation as measured by a negative evoked potential component (NI) and increased gamma band phase locking associated with VH in schizophrenia, consistent with an underlying visual cortical hyperexcitability and reduced responsiveness to external visual stimulation in this subgroup.^102^ Indices of hyperexcitability and reduced responsiveness have also been found in PD (a delayed P100 evoked potential component)^103^ and post-lysergic acid diethylamide VH (occipital theta coherence).^104^ The electrophysiology of VH capture is consistent with brain imaging studies in suggesting activation of visual association cortex as measured by coherence^105^ or theta/alpha desynchronization.^106^

## Comparative Psychopathology

What are the implications of the similarities and differences between VH in psychosis spectrum, eye and neurodegenerative disease outlined above? It is clear that there is an absence of evidence in many areas, and that direct comparative work is required. VHs in psychosis most closely resemble VH in neurodegeneration, and the association of auditory and VHs in these conditions suggests the 2 modalities of hallucination share a common pathophysiological mechanism. Dysfunctions in attentional and executive/top-down mechanisms are also common in both neurodegenerative and psychotic conditions, as are occipital cortex and HC involvement during hallucinations.

However, the differing predominance of auditory and visual modalities in neurodegenerative disease and psychosis, the phenomenological differences at the levels of emotional reactions and appraisals, and the differing dysconnectivity and visual processing profiles suggest there are also important differences. Perhaps the same pathological mechanism has differential effects on the visual and auditory systems in psychosis and neurodegenerative disease, depending on the presence/absence of specific co-occurring sensory dysfunctions. Such comparative insights have potentially important implications for treating VH as they provide a rationale for importing approaches found effective in one clinical context to another.

## Treatment Approaches

### Cognitive Behavioral Therapy

Cognitive behavioral therapy has an established, though modest, evidence base for the treatment of psychosis (referred to as CBTp). However, evidence for its use in VH rests mainly on case studies.

Recently, O’Brien and Johns^107^ described an individual who had visions of snakes and who held beliefs that she was at risk of harm from these snakes. CBT that included a graded exposure approach to help reduce fear and escape reaction showed some benefit which was maintained at 3 months. Whether this is helpful with other visual experiences is unclear, although studies show that the appraisals and beliefs should be a core target for treatment.^70,108^

Collerton and Dudley^109^ developed a model drawn from a cognitive model of auditory hallucinations and panic and which targets appraisal and reactions to VH. Given that avoidance, escape and safety-seeking behaviors are common but unhelpful strategies, the aim of treatment is for people to recognize that they are safe, and that the fear may be real but the danger is not. The therapy includes psychoeducation about VH, reality testing and normalization, as well as understanding of triggers. Imagery exposure and transformation strategies are used to attempt control over the experience. A case series (under review) describes the use of this treatment approach in 4 people with psychosis with distressing VH.

Overall, evidence for the effectiveness of CBT for VH is limited. The treatment of VH may be improved by addressing trauma and stress as a key feature of VH presentation, and with the development of sensitive and specific outcome measures that match the goals of treatment.

### The Pharmacological Management of VH

There is limited evidence for specific pharmacological treatments for VH in psychosis. Antipsychotics are typically used, but some studies have reported that VHs were not linked to treatment effects^28,110^ or even that VHs may be a marker of neuroleptic resistance.^10^ Clozapine has been reported as effective for VH in PD,^111^ but studies in schizophrenia are lacking.

The literature drawn from organic disease suggests the different approaches that might be considered. For example, for individuals with visual impairment, treatment of the eye condition can reduce the risk of VH.^112^ The case report literature has highlighted a range of medications that may help reduce VH, but there is currently a lack of clinical trial data. For example, drugs increasing cholinergic activity are thought to reduce the risk for VH. The AchE-I (rivastigmine, donepezil, and galantamine) are beneficial when treating behavioral and psychological symptoms of dementia^113^ and PD^114^ and have been reported to help in schizophrenia.^114^ Other drugs have been used in organic conditions (antidepressants, memantine, or anticonvulsants) but have not been examined in psychosis.

In sum, there is a lack of systematic studies, but this overview suggests new possibilities for treating VH in psychosis.

## Conclusion

Altogether, this article reviewed the available evidence on the prevalence, phenomenology, clinical characteristics, assessment methods, cognition, brain imaging, electrophysiology, and treatment approaches for VH in the psychosis spectrum.

There are significant potential benefits to making direct comparisons of VH in organic and psychotic illness. These include informing our understanding and theoretical models of VH in schizophrenia and across diagnostic categories, informing hypothesis-driven questions for research, and suggesting new possibilities for therapeutic interventions.

This is the first review of VH in psychosis to incorporate data using different methodological perspectives and different population groups. One open question is whether VHs in psychosis have the same pathological mechanisms as VH in eye or organic conditions. From the evidence, it is clear that they are not as closely associated as they might appear. They show some similarities in clinical presentation and form, and in cognitive mechanisms, but differences in age, emotional content and appraisal, pathological profile, and neuroimaging findings might argue for different causal pathways. There is also little evidence one way or the other on whether visual perception in psychosis is as impaired as it is in other disorders.

This review also aided our understanding of hallucinations across different modalities, and particularly auditory hallucinations. As seen above, auditory hallucinations and VH frequently co-occur, although not necessarily simultaneously. One possibility suggests a set of core pathological mechanisms in addition to specific modality-dependent dysfunctions. Alternatively, the same pathological mechanism has differential effects on the visual and auditory systems in psychosis and neurodegenerative disease, depending on the presence/absence of specific co-occurring sensory dysfunctions. Clearly, direct comparisons are needed testing the compatibility of theoretical models developed in the 2 modalities of hallucinations (see key future directions in table 4).

**Table 4.** Future Directions

	Future Studies Should Seek to Pursue the Following Research Questions
i)	What is the frequency of VH in psychosis, as assessed using prospective, rigorous, and detailed investigations? What is the relationship of VH with hallucinations in other modalities (eg, temporal relationship, similarities in content and emotional themes)?
ii)	Are “fused” multimodality hallucinations characteristic of schizophrenia or do they occur in other disorders? Is there a close relationship between VH and voices in fused hallucinations, such that there is a match between the vision and the verbal output? Is the acoustic information closely linked to the speaker’s characteristics (sex, age, emotional characteristics)? Are there reports of fused hallucinations of animals (eg, dogs barking), and environmental scenes (eg, car with engine running, water falls), in addition to fused hallucinations of persons (person talking)?
iii)	What are the similarities and differences in the VH profile of individuals with psychosis compared with other psychiatric/neurological conditions at the level of phenomenological characteristics, risk factors, triggers, cognition, psychology, social and brain factors, as assessed using direct comparisons?
iv)	Are the same brain regions and networks activated during VH in psychosis also activated during VH in other conditions? Are the changes in brain structure, function, and connectivity predisposing to VH the same across different conditions?
v)	What are the mechanisms involved in hallucinations in different modalities? Is there a set of core pathological mechanisms in addition to specific modality-dependent dysfunctions? Alternatively, is there one pathological mechanism that has differential effects on visual and auditory systems?
vi)	What is the compatibility of verbal (language based) models of auditory hallucinations which focus on deviations in language areas, with models of *visual* hallucinations?
vii)	What are the mechanisms by which distress and negative mood contribute to both visual and auditory hallucinations?
viii)	What is the predictive value of non-clinical VH (especially for those first observed in childhood/adolescence), as assessed using longitudinal epidemiological study designs?
ix)	Are there different subtypes of VH and which type is most often associated with poorer prognosis and functioning?
x)	Research into the treatment for VH in individuals requiring care is urgently needed, examining the efficacy of existing and new interventions in large samples

*Note*: VH = visual hallucination.

## Funding

F.W. is supported by a National Health and Medical Research Grant (634329) and funding from North Metro Health Service Mental Health (Graylands Hospital).
